# Impact of high-density lipoprotein 3 cholesterol subfraction on periprocedural myocardial injury in patients who underwent elective percutaneous coronary intervention

**DOI:** 10.1186/s12944-018-0670-3

**Published:** 2018-02-02

**Authors:** Kazuhiro Harada, Ryosuke Kikuchi, Susumu Suzuki, Akihito Tanaka, Toshijiro Aoki, Naoki Iwakawa, Hiroki Kojima, Kenshi Hirayama, Takayuki Mitsuda, Takuya Sumi, Yosuke Negishi, Hideki Ishii, Toyoaki Murohara

**Affiliations:** 1 Department of Cardiology, Nagoya University Graduate School of Medicine, 65, Tsurumai-cho, Showa-ku, Nagoya, 466-8550 Japan; 2 Department of Medical Technique, Nagoya University Hospital, Nagoya, Japan

**Keywords:** High-density lipoprotein cholesterol, High-density lipoprotein 2 cholesterol, High-density lipoprotein 3 cholesterol, Periprocedural myocardial injury, Percutaneous coronary intervention

## Abstract

**Background:**

Periprocedural myocardial injury (PMI) is a major complication of percutaneous coronary intervention (PCI) and is associated with atherosclerotic coronary plaque and worse clinical outcomes. High-density lipoprotein cholesterol (HDL-C) is a protective factor for cardiovascular disease. However, the role of HDL-C subfractions, such as HDL2 cholesterol (HDL2-C) or HDL3 cholesterol (HDL3-C), in cardiovascular disease remains unclear. The purpose of the study was to investigate the relationship between HDL2-C and HDL3-C subfractions and the incidence of PMI in patients who underwent elective PCI.

**Methods:**

We enrolled 129 patients who underwent elective PCI for stable angina pectoris. PMI was defined as an increase in high-sensitivity troponin T levels > 5 times the upper normal limit (> 0.070 ng/mL) at 24 h after PCI. Serum HDL-C subfractions (HDL2-C and HDL3-C) were assessed using ultracentrifugation in patients with and those without PMI.

**Results:**

HDL3-C levels were significantly lower in patients with PMI than in those without (15.1 ± 3.0 mg/dL vs. 16.4 ± 2.9 mg/dL, *p* = 0.016) and had an independent and inverse association with PMI (odds ratio, 0.86; 95% confidence interval, 0.74–0.99; *p* = 0.038). When divided by the cut-off value of HDL3-C for PMI (14.3 mg/dL), the incidence of PMI was significantly higher in low HDL3-C patients than in high HDL3-C patients (51.2% vs. 30.2%, *p* = 0.020).

**Conclusions:**

HDL3-C was an independent inverse predictor of PMI in patients who underwent elective PCI.

## Background

According to past epidemiological data, high-density lipoprotein cholesterol (HDL-C) has a strong inverse relationship with cardiovascular disease (CVD) and is an independent predictor of CVD [1–3]. Therefore, HDL-C has been attracting considerable attention as a preventive target to address residual CVD risk next to low-density lipoprotein cholesterol (LDL-C). However, drugs that increase HDL-C levels such as fibrates, niacin, or most of cholesteryl ester transfer protein (CETP) inhibitors have been proven difficult to show improved CVD outcomes beyond those achieved with statin therapy [4–9]. Thus, it is valuable to reconsider both HDL-C absolute concentrations and subfractions. HDL-C is classified into subfractions by size, density and composition, and it has been reported various methods for measurement of HDL-C subfractions. Several reports have suggested a relationship between HDL-C subfractions and CVD or atherosclerosis [10–12]. However, because of its diversity and complexity, the association between HDL-C subfractions and CVD risk has not been elucidated.

Periprocedural myocardial injury (PMI) is widely known as a complication of percutaneous coronary intervention (PCI), and a previous study showed an association with atherosclerotic coronary plaques such as large burden and lipid-rich plaques [13]. Moreover, a large body of data has demonstrated that PMI is associated with higher mortality rates, even when patients do not develop electrocardiographic changes and symptoms [14–16]*.* Because of the impact of PMI on worse clinical outcomes, the risk management and prediction of PMI is beneficial for clinical practice.

Our hypothesis is that HDL-C subfractions are associated with the incidence of PMI because of their effects to atherosclerosis. HDL2-C and HDL3-C subfractions have been most reported among HDL subfractions in human observation studies or intervention studies by drugs, however the effects of HDL2-C and HDL3-C for PMI remain unclear.

The purpose of the present study was to investigate the relationship between HDL-C subfractions separated into large buoyant HDL2-C and small dense HDL3-C based on their densities after ultracentrifugation, and PMI in patients who underwent elective PCI.

## Methods

### Patient population and study design

**Fig. 1:**
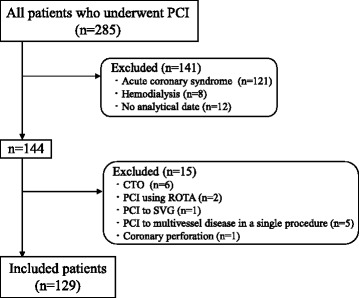
Study flow chart. PCI, percutaneous coronary intervention; CTO, chronic total occlusion; ROTA, rotational atherectomy; SVG, saphenous vein graft; PMI, periprocedural myocardial injury

This study was a retrospective and observational study. We evaluated 285 consecutive patients with documented myocardial ischemia who underwent elective PCI for stable angina pectoris (SAP) at Nagoya University Hospital between February 2015 and August 2016. The key exclusion criteria were acute coronary syndrome (ACS) including unstable angina pectoris (UAP), hemodialysis, lesion characteristics such as chronic total occlusion, saphenous vein graft, and requirement for rotational atherectomy, and PCI for multivessel disease in a single procedure. The details of patient enrollment are shown in Fig. 1, 129 patients were ultimately included in this study. This study was approved by the local ethics committee and conducted in accordance with the ethical principles of by the Declaration of Helsinki. Written informed consent was obtained from all patients.

### Data collection

All patients underwent blood sampling after a 12-h overnight fast. Cardiac enzymes and high-sensitivity troponin T (HsTnT) were measured just before and at 24 h after PCI. Serum HDL2-C and HDL3-C subfractions were measured using ultracentrifugation. Serum samples were adjusted specific gravity with potassium bromide (KBr) (d = 1.063) (a) and without KBr (b) (d = 1.125). They were centrifuged at 42000 rpm for 4-h at 10 °C. After eliminating each 40% of supernatant, HDL2-C and HDL3-C were quantitated as follows: HDL2-C (mg/dL) = [(a) – (b) × 1.54] × 0.6, HDL3-C (mg/dL) = (b) × 1.54 × 0.6 [17].

HsTnT was measured using an electrochemiluminescence immunoassay (Roche Diagnostics, Tokyo, Japan). In this method, the upper normal limit of the reference range was 0.014 ng/mL. The definition of PMI was an increase HsTnT levels of 5 times-over the upper normal limit (> 0.070 ng/mL) at 24 h after PCI [18]. Patients were divided into two groups based on the incidence of PMI.

Hypertension was defined as diastolic blood pressure ≥ 90 mmHg, systolic blood pressure ≥ 140 mmHg, and/or administration of hypertensive medicine. Dyslipidemia was defined as triglycerides ≥150 mg/dL, LDL-C ≥ 140 mg/dL, HDL-C ≤ 40 mg/dL [19] and/or administration of antidyslipidemic medicine. Diabetes mellitus (DM) was defined as having fasting plasma glucose concentration > 126 mg/dL and/or a glycosylated hemoglobin concentration ≥ 6.5% (National Glycohemoglobin Standardization Program) and/or the use of any antihyperglycemic medicine. Current smoking was defined as a declaration of active smoking. The estimated glomerular filtration rate (eGFR) was calculated using the equation for Japanese subjects recommended by the Japanese Society of Nephrology: eGFR (mL/min/1.73m^2^) = 194 × serum creatinine^-1.094^ × age^-0.287^ × 0.739 (if female) [20].

### Percutaneous coronary intervention procedure

The balloon or stent position, size, inflation pressure and time or the use of pre/post-dilation were selected by the operator according to the angiography and conventional intravascular ultrasound findings. PCI success was defined as a reduction of stenosis to < 30% without flow-limiting dissection, occlusion of a side-branch (> 1 mm), or slow-flow/no-reflow phenomenon during the procedure. Dual antiplatelet therapy with aspirin (100 mg/day) and thienopyridine derivatives was administrated to all patients before PCI. Unfractionated heparin (70 U/kg) was received before the procedure and bolus of 1000–2000 U was added every hour if the procedure lasted for > 1 h. The angiograms were reviewed by two experienced observers who were blinded to this study. The observers were cardiologists in clinical group of Nagoya University Hospital.

### Statistical analysis

Continuous values are expressed as mean ± standard deviation for normally distributed variables or as median (interquartile range) for asymmetrically distributed data. Categorical variables are shown as numbers (percentages). Differences in normally distributed continuous values were assessed using Student’s t-test, while those of asymmetrically distributed data were assessed using Mann–Whitney’s U-test. Differences in categorical variables were assessed using the chi-square test. Univariate and multivariate logistic analyses were performed using each parameter to identify the independent predictors of PMI. A *p*-value < 0.05 was considered statistically significant. All statistical analyses were performed with SPSS version 23 (SPSS, Inc., Chicago, IL, USA).

## Results

A total of 129 patients who underwent elective PCI for SAP were enrolled in this study. Of them, PMI was documented in 48 (37.2%). The summarized clinical characteristics are shown in Table 1, while lesion characteristics and PCI procedure details are shown in Table 2.

**Table 1:** Clinical characteristics

Variables	Periprocedural Myocardial Injury		*p* value
	No (*n* = 81)	Yes (*n* = 48)	
Age (years)	68.7 ± 9.7	69.3 ± 10.1	0.75
Male, n (%)	69 (85.2)	38 (79.2)	0.38
Body mass index (kg/m^2^)	23.7 (21.9–26.7)	24.2 (22.4–25.9)	0.95
Current smoking, n (%)	15 (18.5)	13 (27.1)	0.25
Hypertension, n (%)	63 (77.8)	35 (72.9)	0.53
Diabetes mellitus, n (%)	39 (48.1)	20 (41.7)	0.48
Dyslipidemia, n (%)	62 (76.5)	35 (72.9)	0.65
Prior myocardial infarction, n (%)	20 (24.7)	18 (37.5)	0.10
eGFR (mL/min/1.73 m^2^)	69.0 (58.3–77.6)	65.1 (55.6–76.4)	0.37
Total cholesterol (mg/dL)	158 (139–180)	157 (125–186)	0.81
LDL cholesterol (mg/dL)	89 (74–106)	93 (70–113)	0.76
HDL cholesterol (mg/dL)	44.6 ± 10.3	41.0 ± 10.0	0.049
HDL2 cholesterol (mg/dL)	25.0 ± 6.8	23.5 ± 7.4	0.26
HDL3 cholesterol (mg/dL)	16.4 ± 2.9	15.1 ± 3.0	0.016
Triglycerides (mg/dL)	113 (85–163)	119 (93–156)	0.46
Hemoglobin A1c (%)	6.4 (5.9–6.9)	6.1 (5.7–6.7)	0.11
C-reactive protein (mg/L)	1.2 (0.5–3.4)	1.7 (0.8–4.3)	0.25
ACE-I or ARB, n (%)	54 (63.0)	28 (65.1)	0.34
Beta-blocker, n (%)	30 (37.0)	21 (48.8)	0.45
Calcium channel blocker, n (%)	28 (34.6)	19 (39.6)	0.57
Anti-diabetes drugs, n (%)	28 (34.6)	13 (27.1)	0.38
Statin, n (%)	67 (82.7)	40 (83.3)	0.93
Eicosapentaenoic acid, n (%)	6 (7.4)	5 (10.4)	0.55
Ezetimibe, n (%)	2 (2.5)	2 (4.2)	0.59

Data are indicated as means ± SD or median (interquartile range) or number (percentages). *eGFR* estimated glomerular filtration rate, *LDL* low-density lipoprotein, *HDL* high-density lipoprotein, *ACE-I* angiotensin-converting enzyme inhibitors, *ARB* angiotensin receptor blocker

**Table 2:** Lesion characteristics and procedure

Variable	Periprocedural Myocardial Injury		*p* value
	No (*n* = 81)	Yes (*n* = 48)	
Coronary lesion characteristic			
Left anterior descending, n (%)	40 (49.4)	25 (52.1)	0.92
Left circumflex, n (%)	19 (23.5)	9 (18.8)	
Right coronary, n (%)	21 (25.9)	12 (25)	
Left main trunk, n (%)	1 (1.2)	1 (2.1)	
AHA/ACC type B2 or C, n (%)	68 (84.0)	36 (75)	0.14
Procedure			
POBA only, n (%)	10 (12.3)	3 (6.3)	0.27
Direct stenting, n (%)	30 (37.0)	12 (25.0)	0.16
Bare-metal stent, n (%)	28 (34.6)	15 (31.3)	0.70
Drug-eluting stent, n (%)	44 (54.3)	30 (62.5)	0.36
Pre-dilation, n (%)	51 (63.0)	36 (75)	0.16
Post-dilation, n (%)	57 (70.4)	30 (62.5)	0.36
Total stent length (mm)	18 (15–24)	24 (16–28)	0.024
Total inflation time (second)	110 (70–155)	140 (95–205)	0.027
Maximum pressure inflation (atm)	18 (12–20)	16 (12–20)	0.98
Slow-flow phenomenon, n (%)	0 (0)	1 (2.1)	0.19
Side branch occlusion, n (%)	3 (3.7)	1 (2.1)	0.61

Data are indicated as number (percentage) or median (interquartile range). *AHA/ACC* American heart Association/American College of Cardiology, *POBA* percutaneous old balloon angioplasty

The use of statins, eicosapentaenoic acid, and ezetimibe did not differ between patients with and those without PMI. No patients were treated with fibrate, niacin, or probucol. Angiographical residual stenosis, no-reflow phenomenon, or major distal embolism were not observed in this study. Slow-flow phenomenon was observed in one (2.1%) patient with PMI and side-branch occlusion was observed in one (2.1%) patient with PMI and three (3.7%) patients without PMI, but the occurrences were not significant different. HDL-C and HDL3-C levels were significantly lower and total stent length and total balloon inflation time were significantly longer in patients with PMI. There were no significant differences in other characteristics.

Multivariate logistic regression analysis revealed that the HDL3-C levels had an independent and inverse association with the incidence of PMI (odds ratio [OR], 0.86; 95% confidence interval [CI], 0.74–0.99; *p* = 0.038) after adjusting other risk factor for PMI including age, current smoking, DM, eGFR, total stent length and total inflation time (Table 3).

**Table 3:** Logistic regression analysis to identify predictors of periprocedural myocardial injury

Variables	Univariate		Multivariate	
	OR (95% CI)	*p* value	OR (95% CI)	*p* value
Age	1.01 (0.97–1.04)	0.75	1.01 (0.96–1.05)	0.79
Sex, male	0.66 (0.26–1.67)	0.38		
Body mass index	0.98 (0.89–1.09)	0.73		
Current smoking	1.63 (0.70–3.82)	0.26	2.46 (0.87–6.95)	0.090
Hypertension	0.77 (0.34–1.75)	0.53		
Diabetes mellitus	0.77 (0.37–1.58)	0.48	0.65 (0.27–1.58)	0.34
eGFR	0.99 (0.97–1.01)	0.48	0.98 (0.96–1.01)	0.22
LDL cholesterol	1.00 (0.99–1.02)	0.63		
HDL cholesterol	0.97 (0.93–1.00)	0.058		
HDL2 cholesterol	0.97 (0.92–1.02)	0.26		
HDL3 cholesterol	0.86 (0.76–0.98)	0.018	0.86 (0.74–0.99)	0.038
Triglyceride	1.00 (0.99–1.01)	0.16		
C-reactive protein	1.18 (0.90–1.54)	0.22		
Total stent length	1.06 (1.01–1.10)	0.011	1.04 (0.99–1.09)	0.16
Total inflation time	1.01 (1.00–1.01)	0.014	1.00 (0.99–1.01)	0.22

*OR* odds ratio, *CI* confidence interval. Other abbreviations as in Table 1

We also established the cut-off value of HDL3-C using receiver operating characteristic curve (ROC) for PMI and evaluated the incidence of PMI. The cut-off value of HDL3-C was 14.3 mg/dL (area under the ROC curve, =0.61; *p* = 0.028); 86 (66.7%) patients were assigned to high HDL3-C group and 43 (33.3%) patients assigned to the low HDL3-C group. Moreover, we evaluated the combined analysis using the cut-off values of HDL3-C, HDL-C, and LDL-C. The cut-off value of HDL-C was 40 mg/dL, while that of LDL-C was 100 mg/dL, which considered as the conventional values associated with the risk for CVD [21–23]. The sensitivity and specificity of cut-off value were 45.8% and 74.1% in HDL3-C, 66.7% and 45.8% in HDL-C.

**Fig. 2:**
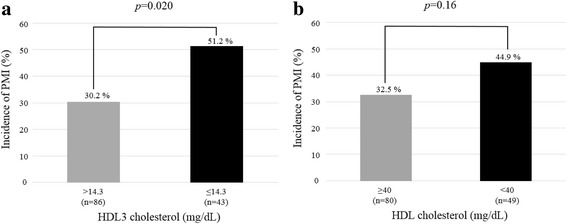
Incidence of PMI in patients with high and low HDL3 cholesterol levels (**a**) or high and low HDL cholesterol levels (**b**). PMI, periprocedural myocardial injury; HDL, high-density lipoprotein

**Fig. 3:**
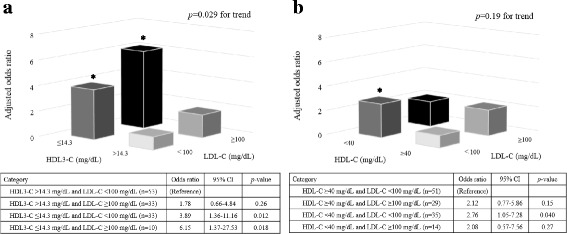
Combination of cut-off value of HDL3-C (14.3 mg/dL) and LDL-C (100 mg/dL) (**a**) and HDL-C (40 mg/dL) and LDL-C (**b**). The model was adjusted for multiple risk factors for PMI including age, sex, body mass index, current smoking, hypertension, diabetes mellitus and eGFR. HDL, high-density lipoprotein; LDL, low-density lipoprotein; PMI, periprocedural myocardial injury; eGFR, estimated glomerular filtration rate; CI, confidence interval. ^*^p < 0.05

The incidence of PMI was significantly higher in patients with a low HDL3-C level (≤14.3 mg/dL) than those with a high HDL3-C level (> 14.3 mg/dl) (51.2% vs. 30.2%, *p* = 0.020) (Fig. 2a), whereas there was no significant difference between the patients with high HDL (≥40 mg/dL) and those with a low HDL (< 40 mg/dL) (Fig. 2b). We evaluated the impact of the combined effect on PMI after adjusting for multiple risk factors for PMI. The highest risk of PMI was revealed when low HDL3-C level was combined with high LDL-C (≥100 mg/dL) (OR 6.15; 95% CI, 1.37–27.53; *p* = 0.018) (Fig. 3a), whereas patients with a low HDL-C and high LDL-C were not at significant risk of PMI (Fig. 3b).

## Discussion

The present study revealed that low HDL3-C levels were significantly associated with the incidence of PMI in patients who underwent elective PCI. Moreover, HDL3-C was an independent and inverse predictor of PMI even after the adjustment of other predictors including total stent length and total inflation time. On the contrary, HDL2-C did not show a significant association with PMI in this population.

Superko et al. examined 80 studies to assess the clinical utility of measuring HDL-C subfractions (HDL2-C and HDL3-C) for CVD risk management [24]. According to that report, only eight prospective studies compared HDL2-C and HDL3-C; of them, four showed an association for both HDL-C subfractions, whereas three showed an association for HDL3-C only, and one showed an association for HDL2-C only. Thus, the assessment of HDL2-C and/or HDL3-C for CVD risk was conflicting and did not reach any consensus. Our results suggest that HDL3-C may be superior to HDL2-C for predicting PMI and may play an important role in reducing PMI risk.

PMI results from procedural complications generally include distal embolization, side-branch occlusion, disrupted collateral flow, and coronary dissection [25]. Furthermore, previous studies have shown that several atherosclerotic coronary plaque characteristics such as large plaque burden, plaque with necrotic tissues and lipid-rich plaque were also associated with PMI [26–28].

Multiple atheroprotective effects of HDL-C including cholesterol efflux, anti-inflammatory, anti-coagulant and antioxidant are well established properties [29]. In a recent study, Rohatgi et al. demonstrated that cholesterol efflux capacity, a key step in reverse cholesterol transport, was inversely associated with CVD [30]. In addition, a previous study suggested that HDL-C could reduce the risk and extent of PMI and predict the patient’s long-term prognosis [31]. Most of HDL-increasing therapy using CETP has not been demonstrated to improve of cardiovascular outcomes [7–9], but considering some CETP inhibitors such as torcetrapib and anacetrapib were reported that the in vitro transfer of cholesteryl esters between HDL3-C and HDL2-C was inhibited [32], it may be needed to assess both HDL-C and HDL-C subfractions. Moreover, as previous evidences demonstrated, in HDL-C subfractions, small dense HDL3-C displays a high cholesterol efflux capacity and possesses anti-inflammatory and antioxidative properties [12, 33, 34]. Therefore, these antiatherogenic properties of HDL3-C might stabilize coronary plaques by reducing the plaque burden, necrotic tissue, or lipid-rich plaque and protecting the myocardium, which may prevent PMI.

According to a previous study, chronic kidney disease is considered a strong predictor of PMI [35], however, in this study, eGFR did not differ between patients with and those without PMI. This discrepancy may have occurred because, unlike in the previous study, we excluded patients undergoing elective PCI for UAP to minimize the factors leading to PMI such as thrombus or vulnerable plaque characteristics.

In this study, we also evaluated the combined effect of LDL-C against HDL3-C or HDL-C. The patients with low HDL3-C and high LDL-C levels were at the highest risk of PMI, whereas those with low HDL-C and high LDL-C levels did not show a significant risk of PMI. These findings may suggest that measuring of HDL3-C subfractions is a more important and valuable screening tool than total HDL-C for predicting the incidence of PMI in clinical practice.

### Study limitations

The present study had several limitations. First, it was conducted in a single center and included a relatively small sample size. Second, we did not evaluate details of HDL2-C and HDL3-C particle subpopulations (HDL2a, −2b, −3a, −3b and -3c cholesterol) or HDL-C functions such as cholesterol efflux, anti-inflammatory and antioxidant properties. Third, although the HDL-C metabolism fluctuates, the metabolic speed or dynamic equilibrium state was not considered in this study. Finally, because low HDL-C levels are often accompanied by increased concentrations of small cholesterol-depleted LDL-C and increased concentrations of cholesterol-enriched triglyceride remnants [36], it was difficult to separate other such associated lipoprotein abnormalities. Thus, a large-scale study is required to confirm our results.

## Conclusions

Our results suggest that HDL3-C level was strongly associated with the incidence of PMI and an independent inverse predictor of PMI in patients who underwent elective PCI.

## Abbreviations

ACS: Acute coronary syndrome
CETP: Cholesteryl ester transfer protein
CVD: Cardiovascular disease
HDL-C: High-density lipoprotein cholesterol
HsTnT: High-sensitivity troponin T
LDL-C: Low-density lipoprotein cholesterol
PCI: Percutaneous coronary intervention
PMI: Periprocedural myocardial injury
SAP: Stable angina pectoris
UAP: Unstable angina pectoris
